# Epidemiological, clinical, and laboratory characteristics of 581 human brucellosis cases in Xinjiang, China

**DOI:** 10.3389/fmicb.2025.1541277

**Published:** 2025-05-07

**Authors:** Bin Luo, Qian Wang, Shuting Yang, Xue Song, Zhiwei Li

**Affiliations:** Clinical Laboratory Center, People's Hospital of Xinjiang Uygur Autonomous Region, Urumqi, Xinjiang, China

**Keywords:** brucellosis, Xinjiang, epidemiology, clinical characteristics, laboratory characteristics

## Abstract

**Background:**

Epidemiological and clinical analyses of brucellosis are crucial for the development of surveillance and case management strategies.

**Methods:**

We analyzed the epidemiological and clinical characteristics of 581 human brucellosis cases in Xinjiang. Demographic characteristics of patients with brucellosis and their clinical manifestations were collected and analyzed.

**Results:**

Among the 581 brucellosis patients, the men-to-women ratio was 2.8:1.0 (428:153); the age was (44.41 ± 16.25) years, ranging from 1 to 83 years, mainly concentrated in the 35–60 age group, accounting for 70.91% (412 cases); the ethnic distribution was mainly Uyghur, accounting for 50.60%; the occupational distribution was mainly farmers, accounting for 43.20%. A total of 186 patients had a clear history of contact with cattle and sheep breeding. Clinical staging was mainly chronic stage patients, accounting for 55.24% (321 cases), and there were 48 cases with complications, mainly pain and fatigue, accounting for 8.26%. The most common laboratory examination characteristics were increased erythrocyte sedimentation rate and increased C-reactive protein level, accounting for 29.09% and 23.06%, respectively, and the blood culture detection rate was low (4.48 %).

**Conclusion:**

Patients with brucellosis in the Xinjiang Uyghur Autonomous Region predominantly comprised middle-aged and young men primarily involved in farming. The principal clinical symptoms include pain and fever; however, the positivity rate of *Brucella* cultures in these patients is low. To minimize the risk of missed diagnoses or misdiagnoses, it is recommended to integrate epidemiological history, clinical manifestations, and laboratory examination results into the diagnostic process to facilitate earlier detection and treatment.

## 1 Introduction

Brucellosis is an important zoonotic disease primarily caused by *Brucella* species (Seleem et al., 2010). The *Brucella* species include *Bcanis, suis, abortus, mellitensis, ceti, ovis, microti, pinnipedialis, neotomae, vulpis, papionis*, and *inopinata* (Deng et al., 2019; Moriyón et al., 2023; Ullah et al., 2024). In animals, *Brucella* infection can cause reproductive failure (such as miscarriage, retained placenta, and inflammation of reproductive organs and membranes), arthritis, and bursitis (Sebzda and Kauffman, 2023; Wang et al., 2024; Moradkasani et al., 2024). Patients with brucellosis have symptoms including recurring fever, headaches, migratory joint pain, weakness, muscle pain, fatigue, loss of appetite, sweating, general discomfort, vomiting, and miscarriage (Głowacka et al., 2018). Some complex complications, such as osteomyelitis, sacroiliitis, septic arthritis, spondylodiscitis, and epidural abscesses, occur in clinical practice. In severe cases, they may cause serious diseases, such as meningitis and encephalitis, and even lead to death (Laine et al., 2023). Approximately 2.1 million new human brucellosis cases occur annually, and Asia and Africa account for most of the global risks and cases (Laine et al., 2023). Brucellosis transmission includes direct contact with infected animals, inhalation of airborne agents, consumption of contaminated products, occupational hazards, intrauterine transmission, and other indirect transmissions (Qureshi et al., 2023).

Brucellosis remains a major public health problem in Xinjiang, China (Shi et al., 2018), and is characterized by a high incidence and complex epidemiological situation. Xinjiang is an epidemic area of brucellosis mainly because of its agricultural practices and close contact between people and livestock, especially sheep and goats, which are common sources of infection. Camels and cattle are infected with brucellosis in Xinjiang (Liu et al., 2024a). *Brucella melitensis* is the most prevalent *Brucella* species in animals and humans in Xinjiang (Sun et al., 2016).

Epidemiological data have revealed that the disease mainly affects middle-aged individuals, especially those engaged in agricultural and animal husbandry activities. A large proportion of cases occur in individuals who have direct contact with infected animals or eat dairy products without high-temperature disinfection (Lai et al., 2017). Although treatment options are available, the chronic rate of brucellosis remains high, and the risk of recurrence is significant. The failure to eradicate *Brucella* is common in Xinjiang and its nomadic regions. The lack of cooperation among health officials, veterinary sectors, policymakers, and farmers has been the primary reason why these endemic countries have failed to control and prevent brucellosis in the past decades (Ali et al., 2024). Other factors, including low socioeconomic levels, weak awareness of brucellosis prevention, and difficulty in early diagnosis, could also explain the difficulty in eradicating *Brucella* (Qureshi et al., 2023). Nevertheless, the clinical management and public health intervention measures must be improved. This study aimed to analyze the epidemiological and clinical characteristics of human brucellosis cases in the Xinjiang region and provide valuable evidence for improving prognosis, reducing the occurrence of brucellosis, and relapse of the disease.

## 2 Methods

### 2.1 Patients

**Figure 1:**
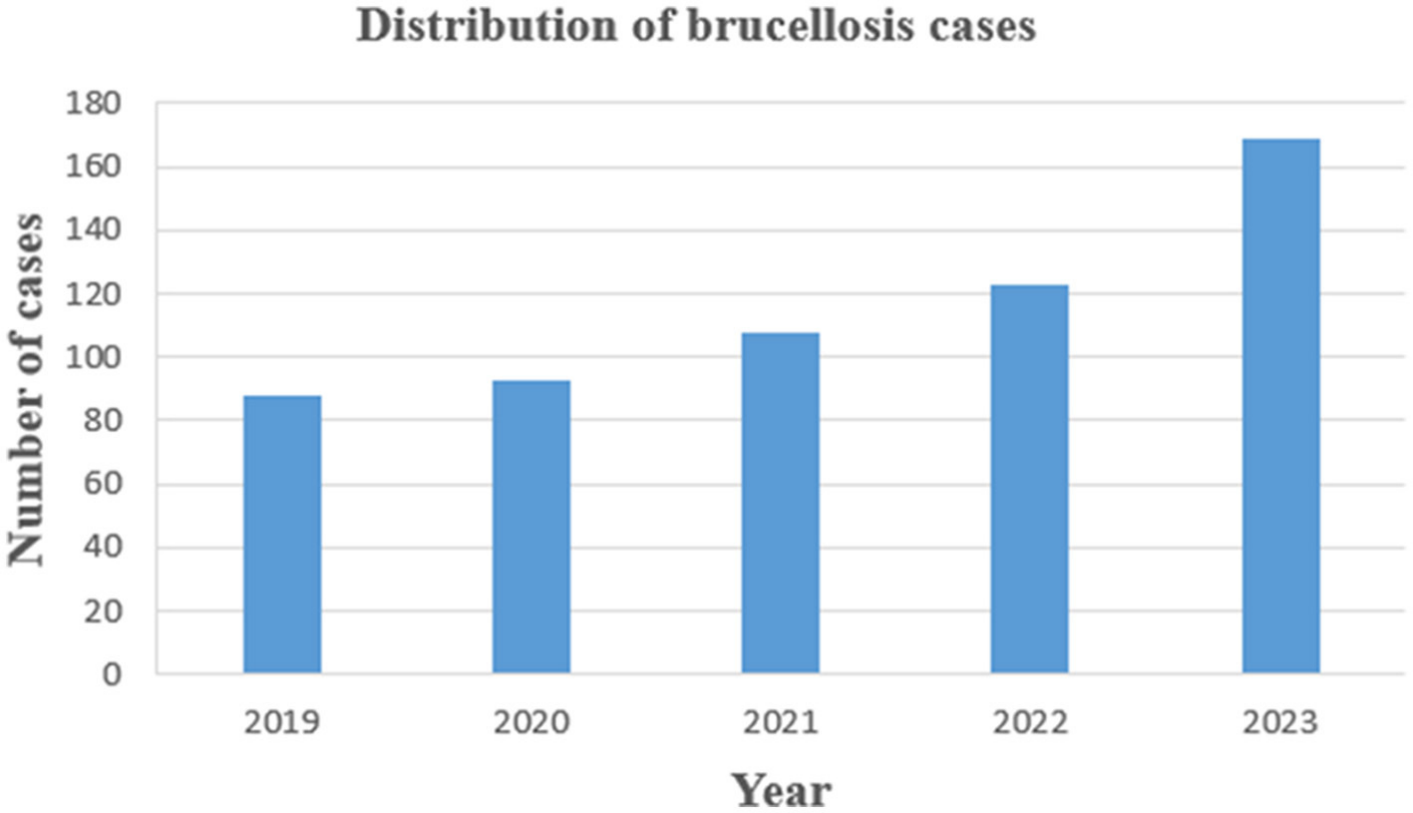
The distribution of the 581 cases by year of diagnosis from 2019 to 2023 in Xinjiang.

**Figure 2:**
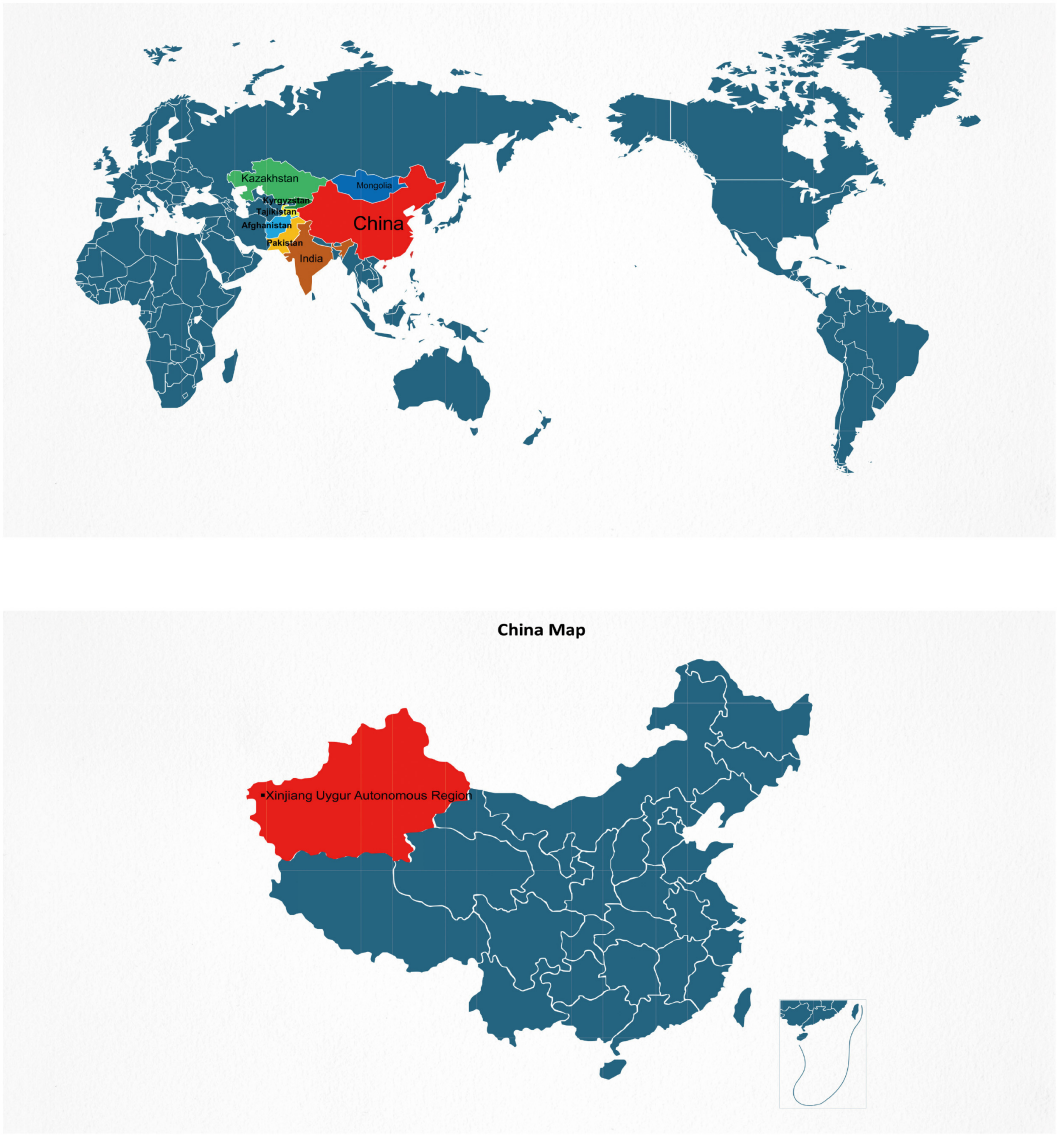
A map showing the location of Xinjiang in China shows its borders with other endemic areas.

This study conducted a retrospective analysis of 581 patients with brucellosis exported from the Information Management System of the People's Hospital of Xinjiang Uyghur Autonomous Region from 2019 to 2023. Outpatient records of research subjects, laboratory indicator test results, clinical treatment, and other materials were collected, and treatment effects were collected through telephone. The distribution of 581 cases by year of diagnosis from 2019 to 2023 is shown in Figure 1. We also included 291 healthy control people for the identifying the relevant factors associated with brucellosis infection. A map showing the location of Xinjiang in China shows its borders with other endemic areas (Figure 2).

### 2.2 Diagnosis of brucellosis

Brucellosis case diagnosis is based on the Chinese health industry standard “Brucellosis Diagnosis” (WS 269-2019, http://www.nhc.gov.cn/wjw/s9491/201905/b109b71e7a624256985b573944b5d292.shtml). Brucellosis was diagnosed based on a combination of epidemiological history, clinical manifestations, and laboratory tests. The diagnostic criteria for brucellosis were (1) epidemiological history, contact with suspected infected animals, consumption of infected meat, or other. (2) Clinical manifestations: fever, excessive sweating, fatigue, and muscle and joint pain. (3) *Brucella* detected in the pathogen culture. (4) Serum agglutination test (SAT) titer ≥ 1:50; or the rose Bengal plate agglutination test (RBT) is positive. The diagnosis of *Brucella* required the presence of criteria (1) or (2), as well as criteria (3) or (4). The clinical staging criteria for brucellosis: 1. acute phase: within 3 months of the course, with a confirmed positive serological reaction; 2. Subacute phase: within 3–6 months of the course, with a confirmed positive serological reaction; 3. Chronic phase: The course exceeds six months and has not healed, with symptoms and signs of brucellosis and a confirmed positive serological reaction. The exclusion criteria were (1) missing personal information or medical records, and (2) positive *Brucella*-specific antibody reactions due to vaccination against brucellosis.

### 2.3 Data collection

Each brucellosis case was comprehensively assessed, including detailed epidemiological contact history, physical examination, and biochemical analysis (blood cell count, routine biochemical parameters, and routine urine analysis). Laboratory values reported in the text and tables refer to the results of the patient's first examination. Patients with complications underwent additional imaging examinations, such as ultrasound, CT, MRI, echocardiography, and scrotal Doppler ultrasound. Relapse was defined as the reappearance of symptoms or a fourfold increase in the SAT titer within 6 months after treatment. All patients with brucellosis received treatments for 6–9 weeks and were followed up for 6 months. For healthy control, the following information was collected: age, sex, ethnicity, occupation, epidemiological history, and complications.

### 2.4 Ethics

The study was conducted in accordance with the principles of the Declaration of Helsinki. The study protocol was approved by the Ethics Committee of the People's Hospital of the Xinjiang Uyghur Autonomous Region. Informed consent was obtained from all patients and patient data were anonymized.

### 2.5 Statistical analysis

The continuous variables were shown as mean ± standard deviations and the categorical variables were presented as number and proportion (%). The t-test and chi-square test were used to analyze differences. Chi-square or Fisher's exact tests were used to analyze the differences in categorical variables; *T* test, One-way ANOVA, and nonparametric tests were used to compare the differences in continuous variables. Statistical significance was set at *P* < 0.05. Data were analyzed using SPSS software (version 21.0, SPSS Inc., Chicago, Ill., USA).

## 3 Results

### 3.1 Demographic and epidemiological characteristics

Among the 581 brucellosis patients, there were 428 men and 153 women, accounting for 73.67% and 26.33%, respectively, with a men-to-women ratio of 2.8:1.0. The age of the participants was 44.41 ± 16.25 years, ranging from 1 to 83 years, mainly concentrated in the 35–60 age group, with 412 cases accounting for 70.91%; followed by elderly patients (60–83 years old), with 104 cases accounting for 17.90%. The ethnic distribution was mainly Uyghur, accounting for 50.60% of the population. No significant differences were found for age, sex, and ethnic distribution between brucellosis group and healthy control group (*P* > 0.05). Brucellosis is an occupational disease affecting farmers and veterinarians and other people (Nedjma et al., 2022). The occupational distribution of this disorder was primarily farming (251 cases, 43.2 %) that was significantly higher than the control group (30.20%, *P* < 0.001).

Of the 390 patients with brucellosis (Table 1), 186 had a relatively clear history of contact with cattle and sheep breeding, and 5 had a history of consuming undercooked meat and dairy products, accounting for 67.13%, 32.01%, and 0.86%, respectively. The ratios were 77.0%, 0.30% and 23.0 in the control group, which was significantly lower than the case group (*P* = 0.012). In terms of clinical staging, chronic stage patients were the main group, with 204 cases (55.24 %); acute and subacute phase patients comprised 321 and 56 (35.11% and 9.64 %, respectively). Complications occurred in 48 (8.26%) patients and 19 (6.50%) in the control group, and there was no significance (*P* = 0.365).

**Table 1:** Demographic characteristics of patients with brucellosis.

**Category**	**Parameter**	**Case (*n*, %)**	**Control (*n*, %)**	***P***
Age groups	1–12 years	40 (6.88)	17 (5.80)	0.116
	12–35 years	25 (4.30)	24 (8.20)	
	35–60 years	412 (70.91)	201 (69.10)	
	60–83 years	104 (17.9)	49 (16.80)	
Sex	Male	421 (73.67)	220 (75.60)	0.474
	Female	153 (26.33)	71 (24.40)	
Ethnicity	Han	119 (20.48)	63 (21.60)	0.337
	Uyghur	294 (50.6)	148 (50.90)	
	Kazakh	100 (17.21)	48 (16.50)	
	Hui	28 (4.82)	9 (3.10)	
	Kyrgyz	12 (2.07)	7 (2.40)	
	Mongolian	10 (1.72)	5 (1.70)	
	Uzbek	1 (0.17)	5 (1.70)	
	Manchu	7 (1.2)	3 (1.00)	
	Other	10 (1.72)	3 (1.00)	
Occupation	Farmers	251 (43.2)	88 (30.20)	< 0.001
	Others	224 (38.55)	60 (20.60)	
	Herdsmen	38 (6.54)	19 (6.50)	
	Workers	39 (6.71)	61 (21.00)	
	Public servants	29 (4.99)	63 (21.60)	
Epidemiological history	No clear epidemiological history	390 (67.13)	224 (77.00)	0.012
	History of consuming undercooked meat and dairy products	5 (0.86)	1 (0.30)	
	History of contact with livestock raising	186 (32.01)	67 (23.00)	
Complications	Yes	48 (8.26)	19 (6.50)	0.365
	No	533 (91.74)	227 (93.50)	
Clinical staging	Acute phase	204 (35.11)	-	-
	Subacute phase	56 (9.64)	-	
	Chronic phase	321 (55.24)	-	

### 3.2 Clinical symptoms and signs

Among the 581 brucellosis patients, the main clinical symptom was pain, with 428 cases (73.67%), mainly lower back pain and joint pain, with 143 (24.61%) and 137 cases (23.58%), respectively; followed by fatigue with 237 cases (40.79%), night sweats with 109 cases (18.76%), nausea and vomiting with 81 cases (13.94%), arthritis with 54 cases (9.29%), palpitations with 19 cases (3.27%), and diarrhea and constipation with 15 cases each (2.58%); the other was asymptomatic, with 59 cases, accounting for 10.15%. There were 387 patients with fever, accounting for 66.61%; there were no significant signs in 82 cases (14.11 %), splenomegaly (56 cases, 9.64 %), lymph node enlargement (32 cases, 5.51 %), hepatomegaly (n = 27, 4.65%), skin rash (12 cases, 2.07 %; jaundice, 5 cases, 0.86 %), or neurological dysfunction, as shown in Table 2.

**Table 2:** Clinical symptoms and signs of brucellosis patient.

**Clinical symptoms**	**Subgroup**	**Number of cases (*n*)**	**Percentage (%)**
Category: clinical symptoms	Asymptomatic	59	10.15
	Fatigue	237	40.79
	Nausea and vomiting	81	13.94
	Palpitations	19	3.27
	Diarrhea	15	2.58
	Constipation	15	2.58
	Night sweats	109	18.76
	Pain	428	73.67
	Arthralgia	137	23.58
	Low back pain	143	24.61
	Abdominal pain	18	3.1
	Back pain	34	5.85
	Headache	65	11.19
	Limb pain	31	5.34
Arthritis	Sacroiliac joint	21	3.61
	Lumbar vertebrae	9	1.55
	Thoracic vertebrae	1	0.17
	Knee joint	10	1.72
	Hip joint	6	1.03
	Upper limb joints	5	0.86
	Other joints	2	0.34
Physical Signs	No significant signs	82	14.11
	Fever	387	66.61
	Hepatomegaly	27	4.65
	Splenomegaly	56	9.64
	Lymphadenopathy	32	5.51
	Joint deformity	3	0.52
	Skin rash	12	2.07
	Jaundice	5	0.86
	Neurological dysfunction	3	0.52
Complications	Meningitis	2	0.34
	Oophoritis	10	1.72
	Orchitis	2	0.34
	Endocarditis	2	0.34
	Pneumonia	28	4.82
	Urinary tract infection	4	0.69

Male patients may also have orchitis, while female patients may show oophoritis. Acute phase patients can have a variety of rashes, some patients can have jaundice, and chronic phase patients show damage to the bone and joint systems.

### 3.3 Laboratory examinations

Among patients with brucellosis, the most significant changes after brucellosis infection were increased erythrocyte sedimentation rate (ESR) and increased C-reactive protein (CRP) levels, with 169 cases (29.09%) and 134 cases (23.06%), respectively. 87 cases (14.99%) had an increased proportion of monocytes/macrophages, leukopenia (42 patients, 7.23%), thrombocytopenia (62 patients, 10.67%), and decreased hemoglobin levels (76 patients, 0.69%). Biochemical examinations revealed an increase in glutamic-pyruvic transaminase and glutamic-oxaloacetic transaminase levels, with 79 cases (13.6%) and 104 cases (17.9%), respectively, and 66 cases (11.36%) with increased alkaline phosphatase levels, with other biochemical projects changing below 10%. Among the 581 patients, only 26 were infected with *Brucella* species, and all of them were infected with *Brucella melitensis*, while the positivity rates of SAT and Tiger Red Agar were high. The specific content could be seen in Table 3.

**Table 3:** Hematological and biochemical findings in brucellosis patients.

**Classification**	**Parameter**	**Number of cases (*n*)**	**Percentage (%)**
Hematological parameters	Leukocytosis	29	4.99
	Leukopenia	42	7.23
	Hemoglobin decreased	76	0.69
	Thrombocytopenia	62	10.67
Liver function tests	ALT > 50(U/L)	79	13.6
	AST > 59(U/L)	104	17.9
	Albumin > 50(g/L)	9	1.55
	Total bilirubin > 22 (umol/L)	19	3.27
	Total bile acids > 10 (umol/L)	32	5.51
	Alkaline phosphatase > 120 (U/L)	66	11.36
Serum enzymes	Creatine kinase > 170 (U/L)	13	2.24
	CK-MB > 5 (ng/ml)	22	3.79
Inflammatory Markers	CRP > 8 (mg/L)	134	23.06
	ESR > 20 (mm/h)	169	29.09
	Procalcitonin > 1 (ng/ml)	58	9.98
Microbiological Tests	Blood culture of *Brucella*	26	4.48
	Tiger Red Agar positive	581	100
	Tube agglutination test (SAT) ≥1:50	245	42.17

### 3.4 Treatment status and results

Of the 581 patients, 379 received first-line treatment (doxycycline combined with rifampicin), 174 received second-line treatment (levofloxacin, rifampicin, or doxycycline), and 28 received third-line treatment (ceftriaxone plus doxycycline or ceftriaxone sodium). The most commonly used treatment plans were levofloxacin, rifampicin, doxycycline, and amoxicillin clavulanate potassium, with 574 cases achieving clinical cure or improvement and seven cases of treatment failure (five patients had drug resistance and two patients did not comply with treatment protocols). During the follow-up period after treatment, 45 patients experienced relapse.

## 4 Discussion

This study retrospectively analyzed 581 human brucellosis cases in Xinjiang between 2019 and 2023 to elucidate the epidemiological characteristics, clinical features, and laboratory findings associated with brucellosis. The results of this study differ from those of previous research (Megged et al., 2016), revealing a higher proportion of chronic cases, which may be linked to the challenges of seeking medical treatment exacerbated by the COVID-19 pandemic. Moreover, restrictions on population mobility may have hindered the treatment of patients in the acute phase Brucellosis is a major zoonotic disorder with high prevalence in animals and humans, especially in the Middle East, Asia, Africa, and South America (Olsen and Palmer, 2014). Brucellosis is endemic in Asian countries. In the Middle East, brucellosis is highly endemic in the majority of countries (Kadir and Gülseren, 2023; Majdil et al., 2024). A study from Iran showed that the incidence rates ranged from 12.07/100,000 to 25.89/100,000 between 2009 and 2017 (Norouzinezhad et al., 2021). The Bekaa District in Lebanon had the highest human brucellosis incidence with 300/100,000 in 2019 (Hassan et al., 2020). The incidence rates in Palestine and Kuwait were 15.32/100,000 and 12.25/100,000, respectively, in 2018. In Saudi Arabia, the incidence rate was 14.17/100,000 in 2017 (Liu et al., 2024b). Among Central Asian countries, data indicated that the incidence rates of human brucellosis in Kazakhstan were 0.038/100,000 in 2019, 12.45/100,000 in Kyrgyzstan in 2018, 2.39/100,000 in Uzbekistan in 2018, 9.71/100,000 in Tajikistan in 2015, and 7.82/100,000 in Turkmenistan in 2016 (Liu et al., 2024b). This disease is also endemic to Pakistan (Tariq et al., 2024); however, brucellosis is highly misdiagnosed and underreported (Jamil et al., 2021). Xinjiang is located on the border with Kyrgyzstan, which is the most endemic region of brucellosis in animals and humans among the five central Asian countries (Kydyshov et al., 2022a,b). Although China is an important trading partner of Kyrgyzstan, the import and export of meat and animals are underdeveloped in both countries (Kydyshov et al., 2022b). A Chinese study reported an upward trend in the incidence of human brucellosis between 2004 and 2021 (Wen et al., 2024). Between 2016 and 2019, the average annual incidence of human brucellosis in China was 3.0/100, 000 individuals (Tao et al., 2021). According to the data from China's National Notifiable Disease Reporting System (Tao et al., 2021), the prevalence of Brucellosis in Xinjiang was 16.3/100,000 in 2019, ranking third in the country. The prevalence rates in Inner Mongolia and Ningxia were 54.4/100,000 and 31.96/100,000, respectively, in 2019 (Wang et al., 2021), which were higher than those in Xinjiang. The annual incidences in the northern region of China ranged from 3.1/100,000 to 28.2/100,000, while the incidence of human brucellosis was lower than 1.0/100,000 in the southern region of China (Tao et al., 2021). The incidence in northern China is significantly higher than that in southern China because animals such as cattle, goats, and sheep are more common in northern China (Lai et al., 2017). Human brucellosis is an ongoing epidemic in the above-mentioned countries, and one undeniable reason is that animal owners are directly involved in animal care and management.

Brucellosis is primarily associated with occupational exposure, dietary habits, and living conditions. Infected animals, along with contaminated food, water sources, and soil, can serve as vectors for the transmission of *Brucella* to humans (Qureshi et al., 2023). The infection rate of human brucellosis is closely correlated with the extent of contact between the sources of infection and the transmission vectors. The occupational risk associated with brucellosis is significant, particularly among individuals who handle sick animals, with farmers representing a major occupational group. Susceptibility to *Brucella* primarily depends on the frequency of contact opportunities (Tao et al., 2021). Brucellosis is an occupational disease that affects farmers, veterinarians, and other people. In this study, 390 patients with brucellosis had no clear epidemiological history. The percentage of patients with brucellosis without a clear contact history with animals infected with *Brucella* was very high. The reasons may be as follows. One, some cases indeed have a contact history with animals infected with *Brucella*, but the brucellosis patients did not realize this, which underestimates the actual percentage of animal contact history. Two, among 390 cases, their occupations were mostly workers, public servants, and others without a history of direct contact with animals infected with *Brucella* in this study. Besides, dairy products are also common of the source of contagion. In the Mediterranean and Latin America, infections have also occurred from consumption of unsterilized goat and camel milk products (Alhussain et al., 2022; Dianelys et al., 2022).

The incidence of *Brucella* infection is higher in males than in females, because men in Xinjiang are more likely to engage in livestock care activities. In our study, 213/428 men and 57/153 women engaged in occupations related to animals. The sex distribution in this study was consistent with that of previous studies (Jia et al., 2017; Shi et al., 2018; Abbas et al., 2025). Young and middle-aged individuals, who constitute the main labor force and frequently come into contact with sick animals, exhibit higher infection rates than those in other age groups. Furthermore, the infection rate of brucellosis is higher in pastoral areas than in urban regions because individuals in pastoral settings interact more frequently with livestock, thereby increasing their exposure to infection. In urban areas, cases are predominantly found among workers in fur, milk, and meat-processing enterprises (Abbas et al., 2025). Among the *Brucella* species, *Brucella melitensis* is the most pathogenic, with sick sheep being the primary source of infection in endemic regions (Shi et al., 2018). *Brucella melitensis* biovars 1, 2, and 3 are highly invasive and pathogenic to humans and animals, easily causing outbreaks and epidemics of human and animal brucellosis, with most presenting as typical clinical symptoms and signs (DelVecchio et al., 2002). This study found that the clinical symptoms and signs of brucellosis primarily include fever, night sweats, fatigue, joint pain, and hepatosplenomegaly [17]. Following infection, *Brucella* can affect multiple organs in the body, leading to corresponding symptoms and signs. Although involvement of the cardiovascular system occurs at a low incidence, it can cause brucellosis-related deaths [18]. In this study, liver function abnormalities were more prevalent among brucellosis patients, with a notable frequency of liver involvement. Patients may exhibit varying degrees of liver function abnormalities, and in some cases, develop liver abscesses. Notably, some patients present with liver function abnormalities as an initial manifestation when seeking medical attention, ultimately leading to a diagnosis of brucellosis.

The clinical manifestations of brucellosis are nonspecific; thus, if clinicians do not prioritize brucellosis in their differential diagnosis and fail to conduct relevant laboratory tests [19], misdiagnosis may occur, resulting in delayed diagnosis and treatment, which can prolong the disease course [20]. In this study, increased erythrocyte sedimentation rate, elevated proportions of monocytes/macrophages, elevated levels of CRP, decreased hemoglobin, and reduced platelet counts were all relatively common laboratory manifestations observed in patients with brucellosis. Several studies have investigated the role of ESR and CRP levels in the diagnosis of brucellosis. Halil et al. showed that the AUCs of CRP and ESR for the diagnosis of brucellosis were 0.635 (sensitivity=57.6, specificity=65.7) and (sensitivity=68.3, specificity=62.9), respectively (Halil and Süleyman, 2020). The cut-off values of CRP and ESR for diagnosing brucellosis complications were > 5.4 mg/L (sensitivity 73.4% and specificity 51.9%) and > 25 mm/h (sensitivity 47.9% and specificity 71.1%) (Shi et al., 2024). A Chinese study of 2,041 human brucellosis cases by Shi et al. revealed that CRP and ESR were not risk factors for an unfavorable prognosis for brucellosis patients (Shi et al., 2018). In their study, the percentages of increased ESR and CRP levels were 69% and 39%, respectively (Shi et al., 2018) which were higher than those observed in our study (CRP=23.06%, ESR = 23.09%). We believe that the higher percentage of acute brucellosis in our study could explain the higher rates of increased CRP levels and ESR observed in the study by Shi et al. (2018). In summary, CRP and ESR are important markers to diagnose brucellosis; however, their sensitivity and specificity are not sufficiently large, which requires other laboratory diagnostic indicators and diagnostic methods to compensate for their shortcomings in diagnosing brucellosis. Currently, the primary basis for laboratory diagnoses relies on various biological agglutination tests. Although bacterial culture positivity is considered the gold standard for the diagnosis of brucellosis, the positive rate was notably low. A diagnosis of brucellosis should be made in conjunction with the patient's epidemiological contact history, clinical manifestations, and laboratory findings for a comprehensive assessment. Notably, other more sensitive alternatives, such as molecular diagnostic methods (e.g., PCR and NGS), were not used in this study, which is a limitation of this study. As this study was retrospective, we could not present the PCR or NGS data. Notably, molecular diagnostic methods (e.g., PCR and NGS) show high sensitivity and specificity for the diagnosis of brucellosis according to data from our hospital in 2024 (data not shown here). Hererin, we recommend that it is necessary to use these molecular diagnostic methods combined with other diagnostic methods to comprehensively diagnose brucellosis. Special attention is warranted when differentiating patients with a history of residing in *Brucella*-endemic areas. In this study, doxycycline combined with rifampicin remained the primary first-line treatment. However, previous studies have indicated that, compared to doxycycline combined with streptomycin, the combination of doxycycline and rifampicin is more prone to treatment failure and relapse (Reza et al., 2012; Solis and Solera, 2012). This may explain the 7.75% (45/581) recurrence rate observed during the six-month follow-up period. Numerous clinical studies have demonstrated that the efficacy of doxycycline combined with streptomycin is comparable to that of doxycycline combined with gentamicin, suggesting that the treatment regimen could be adjusted accordingly for recurrent cases (Roushan et al., 2010; Hasanjani et al., 2006). The combined use of antimicrobial drugs remains the primary treatment method for brucellosis, and an adequate treatment duration is essential to enhance the cure rate and minimize the risk of relapse (Majzoobi et al., 2022).

This study had some limitations. One, the sample size of this study was not very large. Two crucial indices and data were not presented in this study. Third, we address how the characteristics of Xinjiang shed light on regions with similar socioeconomic structures worldwide. Fourth, the challenges and limitations of generalizing our study findings to other populations should be discussed more explicitly. Different levels of government management, socioeconomic status, and educational level may restrict Xinjiang's experience in other regions.

## 5 Conclusion

Given the high incidence of brucellosis in Xinjiang, specific public health measures such as educational campaigns and vaccination programs have been implemented by the government. Although human brucellosis prevention and control have progressed in recent years, brucellosis remains a public health burden in Xinjiang. Central Asian countries surrounding Xinjiang have taken similar health measures to control brucellosis. Continued efforts are essential to enhance public health response measures, improve clinical management, promote vaccination programs including the vaccination of the livestock, and increase community awareness through educational campaigns (Marvi et al., 2018; Laine et al., 2023) to effectively prevent and control this zoonotic disease in these regions. Besides, the primary hosts of brucellosis are cattle (*Brucella abortus*), camel and goats (*Brucella melitensis*), and pigs (*Brucella suis*). etc. For these animal hosts, targeted vaccination is an important measure to effectively prevent brucellosis infection. Implementing surveillance and brucellosis testing is crucial for early detection of this disorder. In Kazakhstan, the incidence of human brucellosis decreased from 2007 to 2019 owing to the implementation of a vaccination program (Liu et al., 2024b). Overall, human brucellosis in these regions is endemic, and brucellosis control and eradication are still difficult because livestock farming may be their main job and only source of livelihood for these brucellosis patients. Their religious beliefs about animals, lifestyle (such as eating habits), and low socioeconomic levels are the key reasons for this (Hikal et al., 2023).

The current situation of human brucellosis in Xinjiang illustrates the intricate interplay between epidemiological, clinical, and socioeconomic factors. As Xinjiang has a high incidence of brucellosis, timely detection and management of the disease are imperative. This study summarizes the clinical and laboratory characteristics of brucellosis in Xinjiang to elucidate its epidemiology, clinical symptoms, and signs in this region. This study aimed to enhance the understanding of brucellosis among clinical and laboratory physicians, reduce instances of misdiagnosis and mistreatment, and provide a foundation for optimizing diagnosis and treatment.

## Data Availability

The original contributions presented in the study are included in the article/supplementary material, further inquiries can be directed to the corresponding author.
